# Exploring the Multiverse of Analytical Decisions in Scaling Educational Large-Scale Assessment Data: A Specification Curve Analysis for PISA 2018 Mathematics Data

**DOI:** 10.3390/ejihpe12070054

**Published:** 2022-07-07

**Authors:** Alexander Robitzsch

**Affiliations:** 1 IPN— Leibniz Institute for Science and Mathematics Education, Olshausenstraße 62, 24118 Kiel, Germany; robitzsch@leibniz-ipn.de; 2 Centre for International Student Assessment (ZIB), Olshausenstraße 62, 24118 Kiel, Germany

**Keywords:** large-scale assessment, item response model, scaling, PISA, multiverse analysis, specification curve analysis, model uncertainty

## Abstract

In educational large-scale assessment (LSA) studies such as PISA, item response theory (IRT) scaling models summarize students’ performance on cognitive test items across countries. This article investigates the impact of different factors in model specifications for the PISA 2018 mathematics study. The diverse options of the model specification also firm under the labels multiverse analysis or specification curve analysis in the social sciences. In this article, we investigate the following five factors of model specification in the PISA scaling model for obtaining the two country distribution parameters; country means and country standard deviations: (1) the choice of the functional form of the IRT model, (2) the treatment of differential item functioning at the country level, (3) the treatment of missing item responses, (4) the impact of item selection in the PISA test, and (5) the impact of test position effects. In our multiverse analysis, it turned out that model uncertainty had almost the same impact on variability in the country means as sampling errors due to the sampling of students. Model uncertainty had an even larger impact than standard errors for country standard deviations. Overall, each of the five specification factors in the multiverse analysis had at least a moderate effect on either country means or standard deviations. In the discussion section, we critically evaluate the current practice of model specification decisions in LSA studies. It is argued that we would either prefer reporting the variability in model uncertainty or choosing a particular model specification that might provide the strategy that is most valid. It is emphasized that model fit should not play a role in selecting a scaling strategy for LSA applications.

## 1. Introduction

Item response theory (IRT) models [1,2] are central to analyzing item response datasets that emerge in educational large-scale assessment (LSA; [3]) such as the (PISA; [4]), the (PIAAC; [5]) or (TIMSS; [6]). The IRT models provide a unidimensional summary of the performance of students on test items in different cognitive test domains. The process of extracting a single summary variable from multivariate item responses is labeled as scaling in LSA.

Interestingly, there is no consensus on which IRT modeling approach should be employed in LSA studies [6,7,8]. This article simultaneously and systematically analyzes the impact of analytical decisions in the scaling model in LSA studies. We use the PISA 2018 mathematics dataset [9] as an example. We follow an approach that integrates results from multiple models because findings from a single model chosen by a particular criterion might not be scientifically sound [10,11]. Moreover, because LSA studies are primarily policy-relevant and less relevant for research, it is vital to investigate whether particular findings are robust regarding different modeling assumptions.

The statistical theory of model uncertainty (or multi-model inference) quantifies the variability in statistical parameters of interest that can be traced back to different model specifications [12,13,14,15]. At its core, the parameter of interest is estimated as a weighted (or unweighted) average of results from multiple models [16,17,18,19,20,21,22]. Many applications can be found in climate research in which researchers have to deal with uncertainty in assumptions about their substantive models [23,24]. This uncertainty is reflected in the variability of findings obtained from different models [25]. A simple example might be reporting uncertainty in weather forecasting of temperature three days or one week ahead.

In the social sciences, the diverse possibilities of model specifications has been addressed with the concepts of multiverse analysis [26,27,28] and specification curve analysis [29,30]. The main idea is to study the variability of findings under the specification of plausible modeling alternatives. This variability should also be reported as an integral part of statistical inference.

In this article, we investigate five important analytical decisions for the scaling model in educational LSA data. First, we consider the choice of the functional form of the IRT model. This choice defines the weighing of each item in the unidimensional summary ability variable [31]. Second, we investigate the treatment of differential item functioning at the country level in the scaling models. Different treatments effectively define at the country level which items should be used for linking a country to an international reference value [32]. Third, the impact of different treatments of missing item responses is investigated. In LSA studies, it is occasionally recommended not to score all missing items as incorrect because missingness might reflect low motivation, which should not be part of the ability variable [33]. Fourth, we discuss the impact of findings due to the choice of particular items in the test. It has been shown that results at the country level could depend on the selected items [34]. Fifth, we investigate the impact of test position effects. It was often empirically shown that items administered at later test positions were more difficult than those presented at earlier test positions. Critically, the impact of test positions also varies across countries which illustrates the dependence of country comparisons on the choice of a particular test design [35].

The rest of the article is structured as follows. In Section 2, we discuss the dataset, the different factors in our multiverse analysis, and the analysis strategy. Section 3 presents the results for the PISA 2018 mathematics dataset. Finally, the paper closes with a discussion in Section 4.

## 2. Method

### 2.1. Data

The mathematics test in PISA 2018 [9] was used to conduct the multiverse analysis. We included 45 countries that did receive the PISA test in a computer-based test administration. These countries did not receive test booklets with lower difficulty items that were specifically targeted for low-performing countries.

In total, 72 test booklets were administered in the computer-based assessment in PISA 2018 [9]. Test booklets were compiled from four clusters of items of the same ability domain (i.e., mathematics, reading, science). In our analysis, we selected test booklets that had two item clusters of mathematics items. As a consequence, students from booklets 1 to 12 were selected. The cluster of mathematics items appeared either in the first and second (booklets 7 to 12) or the third and fourth positions (booklets 1 to 6) in the test.

In total, 70 mathematics items were included in our multiverse analysis. In each of the 12 selected booklets, 22, 23 or 24 mathematics items were administered. Seven out of the seventy items were polytomous and were dichotomously recoded, with only the highest category being recoded as correct. In total, 27 out of 70 items had the complex multiple-choice (MC) format, and 43 items had the constructed-response (CR) format.

In our analysis, 167,092 students from 45 countries were included in the analysis. The sample sizes per country are presented in Table 1 (p. 8). The average sample size of students per country was M = 3713.2. The average number of students per item within each country ranged between 415.8 (MLT, Malta) and 4408.3 (ESP, Spain) and had an average of M = 1120.3.

The IRT scaling models were first fitted on an international calibration sample [36] consisting of *N* = 44,820 students (see Section 2.3). In each of the 45 countries, 996 students were randomly chosen for inclusion in this calibration sample. In a second step, all students within a country were used in the country-wise scaling models to obtain country means and standard deviations.

### 2.2. Analytical Choices in Specification Curve Analysis

In the following five subsections, the definition of five model misspecification factors of our multiverse analysis is described.

#### 2.2.1. Functional Form of the Item Response Model (Factor “Model”)

An IRT model is a representation of the multivariate item response vector $X=(X_{1},\ldots ,X_{I})$ that takes values in ${\left\{0,1\right\}}^{I}$ if *I* denotes the number of items [37,38]. Hence, there are $2^{I}$ different item response patterns. The IRT model assumes the existence of a unidimensional latent variable θ , and item responses $X_{i}$ are conditionally independent of θ . Formally, the IRT model is defined as

(1) $$P\left(X=x;\gamma \right)=\int \prod_{i=1}^{I}P_{i}{\left(\theta ;\gamma_{i}\right)}^{x_{i}}{\left(1-P_{i}\left(\theta ;\gamma_{i}\right)\right)}^{1-x_{i}}f\left(\theta \right)d\theta \;\;\mathrm{for}\;x=\left(x_{1},\ldots ,x_{i}\right)\;,$$

where the item response functions (IRF) are defined as $P_{i}\left(\theta ;\gamma_{i}\right)=P\left(X_{i}=1|\theta ;\gamma_{i}\right)$ and $\gamma_{i}$ denote item parameters. We define $\gamma =(\gamma_{1},\ldots ,\gamma_{I})$ . In principle, IRFs can be nonparametrically identified [39,40,41,42]. Notably, one can view the unidimensional IRT model as an approximation of a true multidimensional IRT model with (possibly strongly) correlated dimensions [43,44,45,46].

In our multiverse analysis, we specify three functional forms of the IRF. First, the one-parameter logistic 1PL (also referred to as the Rasch model; [47]) IRT model is defined as

(2) $$1\mathrm{PL}\;\mathrm{model}:\;P_{i}\left(\theta ;a,b_{i}\right)=\frac{1}{1+\exp(a\left(\theta -b_{i}\right))}\;,$$

where $b_{i}$ is the item difficulty and *a* is the common item discrimination parameter. Second, in the two-parameter logistic (2PL) model [48], the item discriminations are allowed to be item-specific:

(3) $$2\mathrm{PL}\;\mathrm{model}:\;P_{i}\left(\theta ;a,b_{i}\right)=\frac{1}{1+\exp(a_{i}\left(\theta -b_{i}\right))}\;.$$

Third, the three-parameter model with residual heterogeneity (3PLRH) extends to the 2PL model by including an asymmetry parameter $\delta_{i}$ [49,50]

(4) $$3\mathrm{PLRH}\;\mathrm{model}:\;P_{i}\left(\theta ;a_{i},b_{i},\delta_{i}\right)=\frac{1}{1+\exp\left(-{\left\{1+\exp(-\delta_{i}\theta )\right\}}^{1/2}\left(a_{i}\theta +b_{i}\right)\right)}\;.$$

The 3PLRH model has been successfully applied to LSA data and often resulted in superior model fit compared to the three-parameter logistic model (3PL; [48]) that includes a guessing parameter instead of an asymmetry parameter [51,52,53,54]. In this study, we did not include the 3PL model for two reasons, even though the PISA test includes multiple-choice items. It has been argued that the guessing parameter in the 3PL model is not necessarily related to the probability of randomly guessing an item for students that do not attempt to solve an item referring to their knowledge [55,56]. Alternative models might be preferable if the goal is to adjust for guessing effects adequately [55,57]. In a previous study, we demonstrated that the 3PL model did not substantially improve the model fit compared to the 2PL model [54]. In contrast, the 3PLRH model significantly improved the model fit in terms of information criteria [54]. The 3PLRH model is able to account for guessing and slipping effects, as well as for asymmetry in item response functions [53].

In total, the three IRT models, 1PL (factor level “1PL”), 2PL (factor level “2PL”), and 3PLRH (factor level “3PLRH”), are utilized in our multiverse analysis. The 1PL model was used in the PISA study until PISA 2012 [7], while the 2PL model has been employed since PISA 2015 [8,9]. To our knowledge, the 3PLRH has not yet been implemented in the operational practice of any important educational LSA study. The choice of the IRT model in LSA studies has been investigated in [54,58,59,60,61].

#### 2.2.2. Treatment of Differential Item Functioning Based on the RMSD Item Fit Statistic (Factor “RMSD”)

Educational LSA studies compare ability performances across multiple countries. In applications, IRFs are often not invariant across countries. That is, there could exist country-specific item parameters $\gamma_{ig}$ for item *i* in country *g* [34]. This property is also labeled as (country) differential item functioning (DIF; [62,63]). Some restriction(s) on the parameters must be imposed for identification. A popular identification assumption is partial invariance (PI; [64,65]) model in which most of the item parameters for an item *i* are assumed to be equal across countries, while they can differ from a common international item parameter $\gamma_{i}$ for a few countries [5,66,67,68,69,70].

In the operational practice of scaling in educational LSA studies, for each item *i* and each country *g*, a decision is made whether the item parameters are fixed to a common international parameter or they are freely estimated for a country. In practice, the computation of country means and country standard deviations only relies on the invariant items because the linking to the international metric is only conducted on those items. In PIAAC [5] and PISA [8] studies, the root mean square item deviation (RMSD) item fit statistic is used [70,71,72] that is defined as

(5) $${\mathrm{RMSD}}_{ig}=\sqrt{\int {\left(P_{ig}\left(\theta \right)-P_{i}\left(\theta ;\gamma_{i}\right)\right)}^{2}f_{g}\left(\theta \right)d\theta }$$

where $f_{g}$ is the density of the ability variable θ in country *g*.

It has been shown that the RMSD statistic can be effectively used for detecting DIF [5]. Several studies have demonstrated that the RMSD statistic depends on the proportion of misfitting items and the sample size [73,74,75]. Moreover, the distribution of the RMSD statistic for a country depends on the average of uniform DIF effects (i.e., whether DIF is unbalanced or balanced; see [74]).

If the RMSD statistic exceeds a chosen cutoff value, an item is declared to be noninvariant because the country-specific IRF $P_{ig}$ substantially deviates from the model-implied IRF $P_{i}$ . In LSA studies, the cutoff of 0.12 is frequently chosen [5,76]. However, it has been pointed out in the literature that lower cutoff values must be selected to efficiently handle country DIF [72,77,78,79]. In our multiverse analysis, we explore the choice of the three RMSD cutoff values 1.00 (factor level “RMSD100”), 0.08 (factor level “RMSD008”), and 0.05 (factor level “RMSD005”). A rationale for this choice can be found in [78,79]. The cutoff of 1.00 means that all item parameters are assumed to be invariant because the RMSD statistic is always smaller than 1. The RMSD values are obtained from the 2PL scaling in which all item parameters were invariant across countries. In principle, the choice of DIF items will depend on the chosen IRT model. However, to disentangle the factor of the definition of DIF items from other model specification factors in the multiverse analysis, we decided to let the DIF item sets be the same across specifications. Note that the PI approach is practically equivalent to a robust linking approach in which the impact of some items is downweighted (or entirely removed) for a particular country [75,78,80].

#### 2.2.3. Treatment of Missing Item Responses (Factor “Score0”)

In LSA studies, students often do not respond to administered items [81,82,83,84,85,86,87]. Two different types of missing item responses can be distinguished [88]. First, not reached items [89] are missing item responses at the end of a test booklet (or an item cluster). Second, omitted items are missing item responses within the test booklet (or an item cluster) and are no not reached items.

Until PISA 2012, all missing item responses are scored as incorrect. Since PISA 2015, not reached items are treated as non-administered items (i.e., treating it as “NA” in the scaling model), while omitted items are scored as incorrect. Several psychometricians argue that missing item responses should never be scored as incorrect [33,90,91,92,93,94,95,96], while others argue that the treatment of missing item responses is not an empirical question because it should be framed as an issue in scoring, not an issue of missing data modeling [45,88,97,98].

Likely, the choice of the treatment of missing item responses impact on country rankings if the proportion of missing item responses and the missing mechanisms differ between countries [99]. Relatively large differences for some countries have been reported for the PISA study in [88].

In our multiverse analysis, we use three different scoring methods for the treatment of missing item responses. First, all missing item responses are scored as incorrect (factor level “S960”). Second, we scored omitted item responses as incorrect and treated not reached items as non-administered (factor level “S90”). Third, we treat omitted and not reached items as non-administered (factor level “S0”). We have to admit that other proposals in the literature [33,95] will typically lead to results that lie between those from the second and the third approach. However, our three specifications are helpful in deriving bounds for different possible missing data treatments.

#### 2.2.4. Impact of Item Choice (Factor “Items”)

It has been emphasized in generalizability theory that the choice of items should also be included as part of statistical inference, like the sampling of persons [100,101,102,103,104,105,106,107,108]. The uncertainty with respect to items has been quantified as linking errors for trend estimates [109,110,111]. However, a similar error can also be computed for cross-sectional country means [34,112,113]. The reason for the variability in country means with different item sets is the presence of country DIF. That is, performance differences between countries appear to be item-specific. Hence, the country mean is also influenced by the average of country DIF effects for a particular set of chosen items. The variability in country means and standard deviations due to the choice of items can be investigated by using subsamples of items in the multiverse analysis. The half sampling method is a particular subsampling method [80,114] that uses resampling based on half of the sample sizes for determining the variability in estimates. It has been shown that half sampling has superior statistical properties compared to the widely used jackknife method [109].

In our multiverse analysis, we use two item sets. First, we consider the full item set administered in the PISA 2018 mathematics assessment (factor level “All”). Second, we used half of the items in the test (factor level “Part”). In more detail, we used every second testlet (i.e., a group of items with a common item stimulus; see [115]). In the presence of country DIF, we expect that the estimated country means and standard deviations will differ in the two factor levels.

We now formally derive the expected variability due to item choice for our two specifications. Let $\mu_{0}$ be the country mean estimate based on the full item set with *I* items and $\mu_{1}$ be the estimated country mean based on half of the items (i.e., I/2 items). The variance of $\mu_{0}$ and $\mu_{1}$ due to DIF effects is given by

(6) $$\mathrm{Var}\left(\mu_{0}\right)=\frac{\sigma_{\mathrm{DIF}}^{2}}{I}\;\mathrm{and}\;\mathrm{Var}\left(\mu_{1}\right)=\frac{\sigma_{\mathrm{DIF}}^{2}}{I/2}\;,$$

respectively. The DIF variance is denoted by $\sigma_{\mathrm{DIF}}^{2}=\mathrm{Var}\left(e_{ig}\right)$ for DIF effects $e_{ig}$ of item *i* in country *g*, and $\mathrm{Var}\left(\mu_{0}\right)=\sigma_{\mathrm{DIF}}^{2}/I$ is the square of the cross-sectional linking error [112]. In a multiverse analysis, we average across all model specifications. We compute the composite mean $\mu =(\mu_{0}+\mu_{1})/2$ based on the two specifications. Then, we can evaluate the total variance as

(7) $$E\left(\frac{1}{2}{\left(\mu_{0}-\mu \right)}^{2}+\frac{1}{2}{\left(\mu_{1}-\mu \right)}^{2}\right)=\frac{1}{4}E{\left(\mu_{0}-\mu_{1}\right)}^{2}=\frac{1}{4}E{\left(-\frac{1}{I}\sum_{i=1}^{I/2}e_{ig}+\frac{1}{I}\sum_{i=I/2+1}^{I}e_{ig}\right)}^{2}=\frac{\sigma_{\mathrm{DIF}}^{2}}{4I}\;.$$

By comparing (7) with (6), we see that the associated variance with the factor item choice in our multiverse analysis is smaller than the error component associated with $\mathrm{Var}(\mu_{0})$ . The linking error is $\sigma_{DIF}/\sqrt{I}$ , while the square root of the variance of the associated variance component in our multiverse analysis is given by $\sigma_{DIF}/\left(2\sqrt{I}\right)$ (see Equation (7)). Because we report the square roots of variance components in the Results section, we have to multiply the result regarding the multiverse analysis factor “Items” by two to obtain the linking error. It can be shown that considering only half samples of items would result in an unbiased variance component [80,114]. However, in such an approach, the original scaling model that includes all items would not be part of the multiverse analysis, which might be considered a disadvantage.

#### 2.2.5. Impact of Position Effects (Factor “Pos”)

The PISA test involves testing students with a test booklet that lasts two times 60 min of testing time. It is conceivable that student’s test performance can fluctuate in the course of a test. Most likely, performance declines will be observed during the test [116,117,118,119,120]. Items administered at later test positions will typically be more difficult than if they were earlier administered in the test [121,122,123]. Moreover, position effects often differ between persons and, hence, across countries in LSA studies [124,125,126,127,128].

The investigation of position effects in LSA studies is often conducted by including additional latent variables [126,129,130]. In such an approach, the ability variable of interest is defined as the performance at the first test position [35,131,132,133]. If students only got items at the third or fourth test position, the abilities of those students are adjusted and extrapolated to the first test position. Hence, the country means of an ability variable are model dependent.

Consequently, in our multiverse analysis, we study the impact of position effects in a design-based approach. We use three test specifications. First, we considered all students and items at all test positions (factor level “Pos1234”). Second, we used students and items at the first and second test positions in the scaling models (factor level “Pos12”). Third, we used all students and all items at the first test position (factor level “Pos1”). Obviously, the sample size was reduced in the second and the third specification. However, the definition of the ability variable is entirely defined by the test design and, in contrast to the approaches in the literature, is not dependent on a particular scaling model.

### 2.3. Analysis

In total, 3 (scaling models) × 3 (RMSD cutoff values) × 3 (missing data treatments) × 2 (item choice) × 3 (position effects) = 162 models were specified in our multiverse analysis. We declared the reference model as the 2PL model with an RMSD cutoff value of 0.08, scoring only omitted items as incorrect (while treating not reached items as non-administered), used all items for scaling, and the students and items at all four test positions. This approach follows the one employed in PISA 2018 [9].

In each model specification, we scaled the international calibration sample of *N* = 44,820 students for obtaining international item parameters. In the next step, the country mean and country standard deviation were obtained in a separate scaling model for each country in which item parameters were fixed to the international item parameters from the first step except for items whose RMSD values exceed the pre-specified cutoff value. For the country-wise scaling models, student weights were used in marginal maximum likelihood estimation. To enable comparisons across the different model specifications, the ability distributions were linearly transformed such that the total population involving all students in all countries in our study has a mean of 500 and a standard deviation of 100. According to the official PISA approach, standard errors are computed based on the balanced repeated replication (BRR) method [9,114].

For each country, M=162 distribution parameters ${\hat{\gamma }}_{m}$ ( m=1,…,M ) for means and standard deviations are obtained in the multiverse analysis. These parameters are summarized in a multi-model inference [12]. A composite estimate ${\hat{\gamma }}_{\mathrm{comp}}$ based on all model specifications is defined as the equally weighted average

(8) $${\hat{\gamma }}_{\mathrm{comp}}=\frac{1}{M}\sum_{m=1}^{M}{\hat{\gamma }}_{m}$$

Model uncertainty is quantified as the model error (ME) that is computed as the square root of average squared parameter deviations (see [12,54])

(9) $$\mathrm{ME}=\sqrt{\frac{1}{M}\sum_{m=1}^{M}{\left({\hat{\gamma }}_{m}-{\hat{\gamma }}_{\mathrm{comp}}\right)}^{2}}$$

It is interesting to compare the influence of model error (i.e., uncertainty due to different model specifications) with the uncertainty due to sampling of students that is reflected in the error ratio (ER; [54]). The error ratio is defined by

(10) $$\mathrm{ER}=\frac{\mathrm{ME}}{\mathrm{SE}}\;,$$

where SE is the standard error of the composite estimate ${\hat{\gamma }}_{\mathrm{comp}}$ . This standard error is also easily computed with the BRR method because the estimated model parameters for each model specification are available in each replication sample.

It should be noted that we equally weigh all models in the computation of the composite estimator (Equation (8)) and the quantification of variability (Equation (9)). However, such a choice assumes that all model specifications would be considered equally plausible, which has been criticized in the literature [54,134,135]. It might be more legitimate to downweight similar models and upweight models that provide very different results with respect to a target criterion [136,137,138]. Because to our knowledge, almost all of the applications of multiverse and specification curve analysis used equal weights, we also follow this strategy in this article.

Our multiverse analysis varies 5 model specification factors, each having 2 or 3 factor levels. To analyze the importance of each of the factors in model outcomes, we specified a two-way analysis of variance (ANOVA) and computed the extent of explained variance of each of the one-way and two-way factors (see also [139]). In a preliminary analysis, it turned out that no higher-order interactions than two are required because no non-negligible amount of variance was explained by additional higher-order factors. For ease of comparability with standard errors due to sampling of students, we report the square root of the variance component (SRVC; i.e., a standard deviation) for each factor (see also [140,141]). Note that we computed the ANOVA model separately for each countries and averaged the variance components across countries before taking the square root to obtain the standard deviations for each factor.

We used the statistical software R [142] in all computations. The R package TAM [143] was used for determining the RMSD statistic from the 2PL model, assuming international item parameters obtained from the calibration sample. The xxirt() function in the R package sirt [144] was used for estimating all scaling models. Graphical visualization of the multiverse analyses was presented using the default plot taken from specification curve analysis [29] in the specr [145] package.

## 3. Results

Table A1 in Appendix B The estimated common item discrimination *a* in the 1PL model was 1.273. The average of the item difficulties $b_{i}$ was 0.43 (SD = 1.47). In the 2PL model, the item discriminations $a_{i}$ had an average of 1.43 (SD = 0.54). The harmonic mean of the item discriminations was slightly lower at 1.32. The item difficulties $b_{i}$ had a mean of 0.60 (SD = 1.73). Interestingly, the correlations between the item discrimination and the item difficulty in the 2PL model was relatively large with *r* = 0.60. The descriptive statistics of the estimated item parameters in the 3PLRH model are for item discriminations $a_{i}$ : M = 1.00, a harmonic mean of 0.93, SD = 0.38; for item difficulties $b_{i}$ : M = 0.40, SD = 1.26; and the asymmetry parameter $\delta_{i}$ : M = 0.31, SD = 0.78. Like in the 2PL model, item discriminations and item difficulties were strongly correlated (*r* = 0.57), while the other two correlations were less substantial ( r(a,δ) = 0.33; r(b,δ) = −0.12).

In Table 1, the results of the ANOVA of the multiverse analysis for country means and country standard deviations in PISA 2018 are presented. Square roots of variance components (SRVC) of factors are displayed in Table 1.

For the country mean and standard deviation, it turned out that the position effect factor (“Pos”) explains most of the total variance in the multiverse analysis. For the country mean, the DIF treatment (“RMSD”) is based on the chosen RMSD cutoff value and the missing data handling (“Score0”). While the chosen IRT scaling model (“Model”) had the least influence on country means, its impact on SRVC was much larger. The two-way interactions in the ANOVA model were less important. Hence, only square roots of variance components for main effects in the ANOVA are reported at the level of countries in the next tables.

In Table 2, the results of the multiverse analysis of PISA 2018 mathematics for μ are presented. For example, Austria (AUT) had a country mean of 508.7 (SE = 3.20) in the reference scaling model. The country means for Austria in the 162 model specifications ranged between 503.6 and 514.8 with an average of M = 509.7. The variability is reflected in the computed model error of ME = 2.97. Hence, model uncertainty has almost the same importance as sampling error which is reflected in the error ratio ER = 0.93. Interestingly, most of the variability in Austria’s country means can be attributed to the DIF treatment based on different RMSD cutoff values (SRVC = 2.34), followed by position effects (SRVC = 1.50).

The variability in the country means across countries was very similar for the reference model (M = 500, SD = 33.37) and the composite estimator across models (M = 500, SD = 33.34). At the level of countries, the model error ranged between 1.22 (FIN) and 5.74 (BRN) with an average value of 3.05 (SD = 1.05). The distribution of the error ratio ER across countries indicated that model uncertainty was (on average) of similar importance like standard errors (M = 1.12), while it substantially varies across countries (SD = 0.47, Min = 0.51, Max = 5.74). These findings imply that there could be good reasons to include the component of model uncertainty in statistical inference.

**Figure 1:**
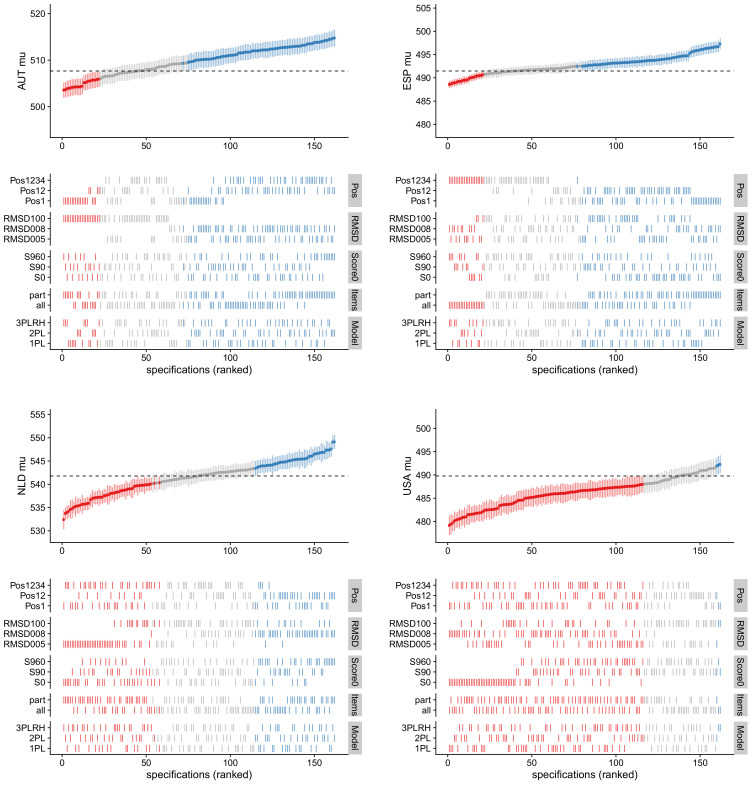
Graphical visualization of multiverse analysis involving M=162 models for country means μ for countries Austria (AUT; **upper left** panel), Spain (ESP; **upper right** panel), Netherlands (NLD; **lower left** panel), and USA (**lower right** panel). The dashed line corresponds to the value from the reference model. Country means colored in blue, gray, or red indicate that they are larger, similar, or smaller than the reference value, respectively.

In Figure 1, the country means for four countries Austria (AUT), Spain (ESP), the Netherlands (NLD) and USA are displayed as a function of factors in the multiverse analysis. These four countries were intentionally chosen to illustrate that the factors in the multiverse analysis have country-specific impacts on their means. Country means that differs from the reference value by at least 0.5 times a standard deviation of a corresponding model are displayed in red or blue lines, respectively. We do not use confidence intervals for inference in Figure 1 because the estimates are strongly dependent across models, and model error is practically uncorrelated with sampling error. That is, model uncertainty constitutes an additional source of uncertainty that is, at least in large sample sizes, unrelated to sampling uncertainty.

For Austria (AUT; ME = 2.97, ER = 0.93; upper left panel in Figure 1), Table 2 indicated that position (“Pos”: SRVC = 1.50) and the RMSD cutoff (“RMSD”: SRVC = 2.34) were the most important factors for the country mean in the multiverse analysis. It can be seen that low country means are obtained for model specifications that involve “RMSD100”. This specification corresponds to the scaling model in which all items were assumed to be invariant. In contrast, specifications with RMSD cutoff values of 0.08 (“RMSD008”) or 0.05 (“RMSD005”) resulted in higher country means for Austria. These specifications allow for some noninvariant items. Critically, the noninvariant items do not contribute to the linking of Austria to the common international metric, which possibly explains difference between the factor levels of “RMSD”. Moreover, if only students and items at the first test position (“Pos1”) were included in the analysis, country means were lower on average compared with the overall mean of M = 509.7 across all model specifications in the multiverse analysis.

For Spain (ESP; ME = 1.91, ER = 0.93; upper right panel in Figure 1), position effects (SRVC = 1.40) were the most important factor. Model specifications that included all four test positions resulted in lower country means (“Pos1234”) than those that included only the first (“Pos1”) or the first and the second test position (“Pos12”). Interestingly, the lowest country mean was obtained if all items were used in combination with RMSD cutoff values of 0.08 and 0.05, resulting in an elimination of some items from linking for Spain.

For the Netherlands (NLD; ME = 3.50, ER = 1.29; lower left panel in Figure 1), the RMSD cutoff value for the treatment of DIF (“RMSD”) had the largest impact (SRVC = 2.61), followed by test position (“Pos”; SRVC = 1.36) and missing data treatment (“Score0”, SRVC = 1.23). The country means for the Netherlands were lowest when the most strict RMSD cutoff value of 0.05 was applied (“RMSD005”). Moreover, if only the first (“Pos1”) or the first and second (“Pos12”) test positions were used in the analysis, country means in the different model specifications were larger on average than the country means based on all four test positions (“Pos1234”). Finally, country means were larger on average if all missing item responses were scored as incorrect (factor level “S960” for the factor “Score0”).

For the USA (USA; ME = 0.90, ER = 0.90; lower right panel in Figure 1), the missing data treatment (“Score0”) had the largest impact on country means (SRVC = 2.32). Country means were lower on average if all missing items were scored as non-administered (“S0”). In contrast, country means for the USA were larger if all missing items were scored as incorrect (“S960”) or only omitted items were scored as incorrect (“S90”).

In Table 3, the results of the multiverse analysis of PISA 2018 mathematics for σ are presented. The average model error (ME) across countries was 2.98 (SD = 1.13) and ranged between 1.27 (Spain; ESP) and 5.55 (The Netherlands, NLD). The error ratio (ER) for country standard deviations was 1.45 on average (SD = 0.50; Min = 0.74, Max = 3.05) and slightly larger than the ER for country means. This means that model uncertainty induced more variability in standard deviations than sampling uncertainty due to the sampling of students (see also findings in [54]).

**Figure 2:**
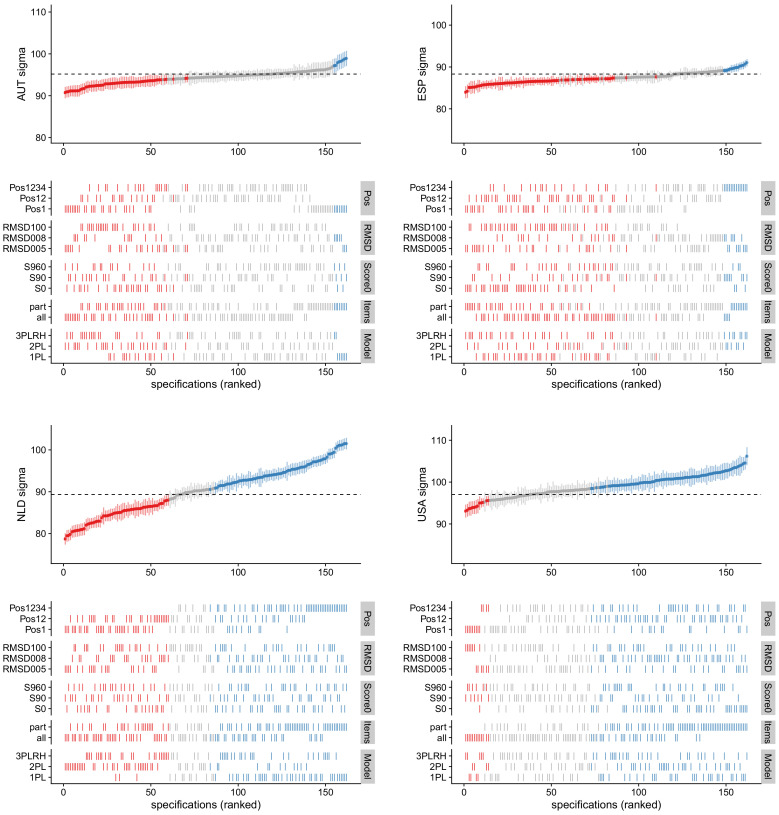
Graphical visualization of multiverse analysis involving M=162 models for country standard deviations σ for countries Austria (AUT; **upper left** panel), Spain (ESP; **upper right** panel), Netherlands (NLD; **lower left** panel), and USA (**lower right** panel). The dashed line corresponds to the value from the reference model. Country standard deviations colored in blue, gray, or red indicate that they are larger, similar, or smaller than the reference value, respectively.

In Figure 2, the country standard deviations for four countries Austria (AUT), Spain (ESP), the Netherlands (NLD) and USA are displayed as a function of factors in the multiverse analysis. The model errors for Austria (ME = 1.60) and Spain (ME = 1.27) were smaller than for the Netherlands (ME = 5.55) and the USA (ME = 2.60).

The variability in standard deviations for the Netherlands (NLD; lower left panel in Figure 2) was particularly large (M = 90.2, Min = 78.7, Max = 101.5). Test position (“Pos“; SRVC = 3.75), choice of the IRT model (“Model”; SRVC = 2.96), and item choice (“Items”; SRVC = 1.80) had the largest impact. The country standard deviations computed on all four test positions (“Pos1234”) were larger than those obtained from the first (“Pos1”) or the first and the second (“Pos12”) test positions. The standard deviations based on the 1PL model were larger on average than those obtained with the 2PL or the 3PLRH models.

## 4. Discussion

Our study illustrates that model uncertainty (i.e., model error) cannot be neglected in outcomes of educational LSA studies such as PISA. It was shown that model error was more pronounced in country standard deviations than in country means. Discussions about model specifications in the literature often focus on the influence of country means or country rankings. This might have led to false impressions that particular modeling choices were less consequential.

It turned out that all five considered specification factors in our multiverse analysis had an impact on either country means or standard deviations or both statistics. Test position impacted the mean and the standard deviation. Interestingly, the DIF and the missing item response treatment mainly affected the country mean more than the standard deviation. At the same time, the choice of the IRT model strongly influenced the standard deviation (see also [54]).

Particular model specification choices differentially impact the mean or the standard deviation of a country. For example, the choice of different RMSD cutoff values depends on the proportion of DIF items in a country. Moreover, the missing item response treatment will mainly affect countries with relatively low or high missing proportions compared to the average proportion of all countries. We studied the model error and the error ratio for quantifying the country-specific model uncertainty in our multiverse analysis.

If all model specifications are plausible, model uncertainty can be ignored and considered part of the statistical inference in country comparisons in educational LSA studies. By varying different model specifications, different assumptions about model generalization are made. This perspective was taken in a sampling model of validity [146,147].

In [45], we argued that the computation of statistics for the latent variable θ (i.e., the ability variable) should be mainly motivated by design-based considerations. We think that particular specification choices are preferable for the five considered factors in our multiverse analysis. We will discuss our preferences in the following.

First, for the test position, we think that the test design should be defined a priori. We do not think that it is a threat to validity because country rankings can change if the first two or all test positions were used in an analysis. The computed ability in a longer test of 120 min testing time represents a different test situation than in a test that only involves 60 min of testing time. A researcher must define how ability should be assessed. Some researchers argue that test position must be disentangled from performance decline that could be due to lower test motivation at later test positions [131]. We do not think that it is useful to define ability independent of test motivation. One could put the argument to the other extreme that average performance should be computed only for one administered item per student at the beginning of the test because the performance on subsequently administered items also depends on test persistence.

Second, we think that the mechanistic inclusion of country-specific item parameters for DIF items based on certain RMSD cutoff values decreases validity because country comparisons effectively only rely on the items that are declared to be non-DIF-items [45,79]. If substantial DIF for an item is detected, researchers must judge whether the DIF truly refers to a bias in measurement for a country. That is, it must be decided whether DIF is construct-relevant or construct-irrelevant [32,63,78]. In the PISA studies until PISA 2012, DIF items were only removed from analysis if technical reasons or explanations for the DIF were found [148,149]. Hence, DIF items for a particular item had international item parameters that were assumed to be invariant across countries, although there is a misfit in some countries. We argued elsewhere [45] that model misfit should be no concern in LSA studies because all IRT models are intentionally misspecified. The model parameters in a selected IRT model receive their sole meaning because of their definition in the likelihood function for deriving summaries of the multivariate item response dataset. Hence, item and model parameters such as country means and standard deviations can be unbiased even if the IRT model is grossly misspecified. Hence, conclusions in the literature that there might be biased country means or standard deviations due to the presence of DIF [70,150] are misplaced.

Third, we believe that missing item responses should always be treated as incorrect in educational LSA studies [45,98]. Otherwise, countries can simply manipulate their performance by instructing students to omit items they do not know [88]. We are also unconvinced that response times are beneficial for obtaining more valid ability measures by downweighing item responses with very fast item responses (see [33] for such arguments). Moreover, proponents of model-based treatments of missing item responses assume that the probability of omitting an item depends on latent variables but not the particular item itself (i.e., they pose a latent ignorability assumption; see [33,85]). It has been shown that this modeling assumption must be refuted by means of model fit [88]. Interestingly, analyses for PISA have shown that the missingness of constructed-response items can be statistically traced back to the fact that students do not know the item. We are also less convinced of the scoring of not-reached items as non-administered since PISA 2015. We think that not-reached items should always be scored as incorrect because ability should be defined on student’s performance for a fixed length, not a test length chosen by the test taker.

Fourth, we have shown that the choice of items can impact country means and standard deviations. We think the uncertainty due to item choice should be included in statistical inference. For cross-sectional and trend estimates [45,112], this concept is labeled as linking error and can be simply determined by resampling techniques of items [54,80]. In this sense, all items should be included in a cross-sectional analysis. With a larger number of representative items for a larger item domain [151,152], the linking error will be smaller. The situation is a bit more intricate for trend estimation in LSA studies (i.e., the trend in country means for PISA mathematics between PISA 2015 and PISA 2018) if the item sets in the two studies differ. Typically, there will be link items that appear in both assessments and unique items that are only administered in one study. In this case, trend estimates computed only on link items might be more efficient than those computed on all items [112] if DIF between countries exists. If the same items were used for trend estimation, stable country DIF effects are blocked because only changes in item performances are effectively quantified in trend estimation. In contrast, the average of DIF effects of unique items and of link items impacts trend estimates if all items were used in the analysis [45].

Fifth, the choice of the IRT model is crucial for defining the impact of items in the ability variable [31,45,54]. Until PISA 2012, the 1PL model was used that equally weighs items in the ability variable. Since PISA 2015, the 2PL model has been utilized that weighs item discriminations that are estimated in the IRT model. We concur with Brennan ([153]; see also [154]) that it is questionable to let a statistical model decide how items should be weighed in the ability variable. The resulting weighing of items might contradict the intended test blueprint composition [31]. Some researchers argue that one should not fit more complex IRT models than the 2PL model, such as the three-parameter logistic (3PL) IRT model. They argue that at most two item parameters can be identified from multivariate data [75] and base their argument on a result of the Dutch identity of Holland [155]. However, Zhang and Stout [156] disproved the finding. Hence, using the 2PL model instead of the 3PL or the alternative 3PLRH model might in LSA studies be rather a personal preference than due to model fit or validity reasons. In typical LSA datasets, item responses are multidimensional, and violations of local dependence are likely found [157,158,159]. We argued above that the chosen unidimensional IRT model must (and will typically) not hold (see also [160]). However, we have shown that for reasons of model fit, the 2PL model must be refuted in the PISA study [54].

Finally, we would like to emphasize that we believe that decisions for model specifications in LSA studies must not be primarily convincing based on research findings, but are selected by purpose. We doubt that that model fit should play a role in reaching a decision. It could be more honest to state that the model specifications of a particular test scaling contractor in LSA studies are part of its role as a player in the testing industry, and every company has its own brands (i.e., IRT models and model specifications). Choices are almost always made by conventions and historical or recent preferences, but the underlying motivations should be transparently disclosed [161]. We doubt that discussions about analytical choice can be resolved by relying on empirical findings.

## Figures and Tables

**Table 1:** Square roots of variance components (SRVCs) associated with factors of the multiverse analysis in a two-way analysis of variance for country mean μ and country standard deviation σ .

	μ	σ
Total	3.05	2.98
Items	**0.89**	**1.13**
Model	**0.60**	**1.48**
Pos	**1.83**	**1.76**
RMSD	**1.52**	**0.91**
Score	**1.37**	**0.84**
Model × Items	0.20	0.35
Model × Pos	0.20	0.42
Model × RMSD	0.36	**0.54**
Model × Score	0.09	0.19
Pos × Items	0.41	**0.69**
Pos × RMSD	0.43	0.44
Pos × Score	0.41	0.29
RMSD × Items	**0.89**	**0.55**
Score × Items	0.22	0.15
Score × RMSD	0.14	0.10

*Note*. Total = standard deviation associated with total variability across models; Items = item choice (see Section 2.2.4); Model = specified IRT model (see Section 2.2.1); Pos = choice for handling position effects (see Section 2.2.5); RMSD = used cutoff value for RMSD item fit statistic for handling DIF (see Section 2.2.2); Score0 = scoring of missing item responses (see Section 2.2.3); Square roots of variance components larger than 0.50 are printed in bold.

**Table 2:** Results of a multiverse analysis for PISA 2018 mathematics for country means.

		Reference Model		Multi-Model Inference					Square Root of Variance Component (SRVC)				
**cnt**	***N***	**Est**	**SE**	**M**	**Min**	**Max**	**ME**	**ER**	**Pos**	**RMSD**	**Score0**	**Items**	**Model**
ALB	2116	439.7	3.39	442.8	434.7	450.1	3.38	1.00	**2.44**	0.46	**1.12**	0.00	**1.00**
AUS	6508	504.4	2.17	505.8	499.6	510.3	2.80	1.29	**2.37**	0.82	0.60	0.82	0.15
AUT	3104	508.7	3.20	509.7	503.6	514.8	2.97	0.93	**1.50**	**2.34**	0.36	0.44	0.38
BEL	3763	523.6	2.39	525.3	522.4	529.4	1.63	0.68	**1.08**	0.23	0.61	0.78	0.34
BIH	2934	415.4	3.21	418.0	405.4	426.8	4.18	1.30	0.73	**1.78**	**2.72**	0.46	**1.52**
BLR	2681	482.5	2.88	478.0	472.9	483.6	2.52	0.88	**1.95**	0.65	0.97	0.35	0.04
BRN	2259	439.0	2.08	430.1	420.0	446.7	5.74	2.75	**3.57**	**2.99**	**2.09**	0.08	**1.16**
CAN	7200	530.4	2.54	527.7	522.8	531.4	1.96	0.77	0.88	0.93	0.59	**1.17**	0.32
CHE	2679	522.7	2.96	524.3	519.5	530.4	2.59	0.88	**1.93**	0.82	0.58	**1.24**	0.33
CZE	3199	510.8	2.70	512.5	507.0	518.6	2.31	0.86	**1.41**	0.85	**1.03**	0.91	0.35
DEU	2482	514.6	3.18	514.1	508.0	518.9	2.39	0.75	**1.23**	**1.11**	**1.25**	0.69	0.35
DNK	3304	522.5	2.30	522.3	515.9	527.8	3.06	1.33	0.81	**2.18**	0.79	**1.53**	0.36
ESP	11855	491.3	1.63	492.7	488.6	497.3	1.91	1.17	**1.40**	0.06	0.45	0.77	0.20
EST	2467	532.7	2.36	534.4	529.7	539.7	1.95	0.83	**1.21**	**1.15**	0.23	0.50	0.22
FIN	2573	514.2	2.40	515.1	512.1	517.4	1.22	0.51	0.25	0.43	0.55	0.08	0.70
FRA	2880	506.0	2.64	506.5	502.4	511.1	2.24	0.85	0.58	**1.49**	0.67	0.99	0.26
GBR	5979	513.3	3.16	516.4	511.7	521.6	1.96	0.62	**1.32**	0.57	0.42	**1.04**	0.17
GRC	2114	458.9	3.74	456.0	450.2	459.7	2.15	0.58	**1.56**	0.83	0.34	0.13	0.23
HKG	2008	564.2	3.74	560.5	546.0	571.9	4.85	1.30	**2.44**	**2.82**	**1.21**	0.80	0.70
HRV	2150	471.1	3.08	470.9	464.0	476.7	3.16	1.03	**2.46**	0.48	0.69	**1.65**	0.19
HUN	2361	492.1	2.77	486.3	476.6	494.9	3.97	1.43	**2.90**	**1.73**	0.13	**1.12**	0.27
IRL	2581	510.4	2.54	502.7	493.7	510.4	3.59	1.41	**2.87**	**1.17**	**1.41**	0.38	0.56
ISL	1485	501.3	2.64	506.6	494.8	517.6	4.83	1.83	**3.68**	**1.35**	**1.60**	0.71	**1.04**
ISR	1944	465.5	4.85	470.0	462.2	478.2	3.57	0.74	**2.20**	**1.38**	**1.88**	0.20	0.94
ITA	5475	496.8	3.00	499.6	494.0	507.8	3.03	1.01	**1.17**	**1.72**	**1.51**	**1.28**	0.29
JPN	2814	539.5	3.08	542.2	537.0	549.1	2.63	0.85	0.09	**1.48**	**1.62**	0.21	0.23
KOR	2200	535.2	3.76	534.3	530.0	541.6	2.66	0.71	0.28	**1.94**	0.26	0.12	0.06
LTU	2265	491.1	2.33	488.7	481.5	495.5	2.99	1.28	**1.87**	**1.16**	**1.12**	**1.31**	0.89
LUX	2407	491.8	2.23	493.6	489.3	499.4	1.89	0.85	**1.28**	0.57	0.79	0.47	0.25
LVA	1751	503.9	2.46	500.5	491.4	508.7	3.34	1.36	**2.23**	**1.81**	**1.23**	0.11	0.69
MLT	1113	481.3	3.77	486.1	480.4	495.9	3.34	0.89	**2.08**	**1.10**	**1.34**	0.99	0.31
MNE	3066	435.6	1.84	441.8	434.4	449.6	3.40	1.84	0.92	**1.17**	**2.29**	**1.33**	**1.10**
MYS	2797	445.4	3.17	441.3	430.2	453.5	5.05	1.60	**2.37**	0.97	**3.76**	0.73	0.56
NLD	1787	542.6	2.71	541.5	532.4	549.1	3.50	1.29	**1.36**	**2.61**	**1.23**	0.52	0.31
NOR	2679	507.5	2.07	511.1	502.5	519.1	3.41	1.64	**1.79**	0.91	**1.58**	**1.82**	0.68
NZL	2821	508.0	2.29	505.3	501.9	509.1	1.60	0.70	0.34	0.93	0.29	0.38	0.31
POL	2577	524.4	3.32	521.6	516.3	526.0	2.29	0.69	**2.04**	0.35	0.20	0.15	0.68
PRT	2730	501.1	2.74	503.3	497.8	513.5	3.46	1.26	0.38	**2.03**	0.95	**2.30**	0.48
RUS	2510	495.4	3.46	497.1	488.9	504.0	3.21	0.93	**1.93**	**1.73**	0.66	**1.15**	0.78
SGP	2201	584.2	2.03	580.3	567.8	592.8	5.21	2.57	**3.01**	**2.95**	**1.31**	0.29	**1.07**
SVK	1904	496.4	3.00	498.9	493.7	506.6	2.90	0.97	**1.54**	**2.04**	0.42	0.58	0.76
SVN	2863	522.0	2.49	523.6	520.0	527.6	1.82	0.73	**1.08**	0.89	0.34	0.14	0.50
SWE	2539	503.4	3.20	511.4	498.9	519.6	4.83	1.51	**2.21**	**2.10**	**2.93**	**1.08**	0.34
TUR	3172	469.1	2.42	462.7	456.1	469.5	2.86	1.18	0.86	**1.63**	**1.90**	0.31	0.28
USA	2218	490.0	3.43	486.3	479.1	492.3	3.08	0.90	0.90	**1.29**	**2.32**	0.37	0.28

*Note*. cnt = country label (see Appendix A); N = sample size; M = composite estimator for multi-model inference (see (8)); ME = model error (see (9)); ER = error ratio defined as ME/SE (see (10)); Items = item choice (see Section 2.2.4); Model = specified IRT model (see Section 2.2); Pos = choice for handling position effects (see Section 2.2.5); RMSD = used cutoff value for RMSD item fit statistic for handling DIF (see Section 2.2.2); Score0 = scoring of missing item responses (see Section 2.2.3); Square roots of variance components larger than 1.00 are printed in bold.

**Table 3:** Results of a multiverse analysis for PISA 2018 mathematics for standard country deviations.

		Reference Model		Multi-Model Inference					Square Root of Variance Component (SRVC)				
**cnt**	N	**Est**	**SE**	**M**	**Min**	**Max**	**ME**	**ER**	**Pos**	**RMSD**	**Score0**	**Items**	**Model**
ALB	2116	87.9	2.03	84.9	75.9	96.2	5.09	2.50	**3.12**	**1.27**	0.67	0.18	**3.11**
AUS	6508	98.2	1.56	95.7	90.3	100.8	2.23	1.43	**1.91**	0.54	0.62	0.18	0.17
AUT	3104	95.5	2.16	94.3	90.7	98.9	1.60	0.74	0.13	0.25	0.24	0.43	0.43
BEL	3763	95.2	1.89	96.4	92.2	100.1	1.73	0.91	0.90	0.22	0.28	0.56	0.89
BIH	2934	87.1	1.78	84.7	74.3	104.0	5.43	3.05	**3.07**	**1.00**	0.96	0.12	**3.72**
BLR	2681	95.0	2.33	100.1	92.7	108.5	3.63	1.56	**3.05**	0.13	0.86	**1.20**	0.41
BRN	2259	96.5	1.73	94.3	88.8	102.5	3.12	1.81	**1.15**	0.55	0.30	0.29	**2.19**
CAN	7200	92.8	1.43	93.2	88.9	97.5	1.88	1.32	0.56	0.24	0.42	0.84	**1.02**
CHE	2679	97.8	2.00	97.3	90.9	101.0	2.00	1.00	**1.24**	0.32	0.55	0.75	0.64
CZE	3199	94.3	1.94	98.0	94.3	103.5	1.75	0.90	0.69	0.70	0.87	0.66	0.56
DEU	2482	97.6	1.73	98.1	93.0	104.0	2.30	1.33	0.63	0.60	0.24	**1.47**	0.43
DNK	3304	86.1	1.78	84.9	77.8	90.3	2.89	1.62	**2.45**	0.72	0.37	0.38	**1.03**
ESP	11855	87.8	1.31	87.4	84.0	91.0	1.27	0.97	0.60	0.48	0.35	0.21	0.25
EST	2467	85.4	1.70	87.6	79.0	95.1	3.49	2.05	0.64	0.31	0.68	**1.96**	**2.30**
FIN	2573	83.2	1.84	85.4	81.0	90.2	2.12	1.15	0.67	0.90	0.68	0.40	0.87
FRA	2880	95.4	2.10	93.1	86.1	96.2	1.87	0.89	**1.17**	0.53	0.44	0.48	0.74
GBR	5979	100.4	1.90	98.7	91.8	105.0	2.83	1.49	**1.69**	0.17	**1.59**	**1.04**	0.42
GRC	2114	91.8	2.45	92.8	86.5	103.4	3.87	1.58	**2.76**	0.83	0.70	0.66	**1.90**
HKG	2008	98.9	2.79	96.8	85.7	107.0	5.03	1.80	**3.45**	**1.92**	0.57	0.02	**2.62**
HRV	2150	86.8	2.54	87.8	82.1	94.8	2.71	1.07	**1.70**	0.56	0.38	0.46	**1.58**
HUN	2361	94.7	2.15	98.7	92.8	106.9	3.52	1.64	**1.36**	**1.35**	0.26	**2.47**	**1.21**
IRL	2581	80.0	1.42	80.1	76.5	84.3	2.11	1.49	**1.31**	0.53	0.28	**1.11**	0.29
ISL	1485	93.5	2.33	93.4	88.2	97.5	2.03	0.87	0.51	0.51	0.43	0.29	0.47
ISR	1944	119.8	3.15	117.9	109.8	128.8	3.97	1.26	**2.05**	0.69	**1.38**	**1.29**	**1.81**
ITA	5475	94.6	2.49	93.9	87.6	97.1	2.11	0.85	0.92	0.34	0.30	**1.59**	0.19
JPN	2814	91.4	2.33	89.1	79.0	97.8	4.33	1.86	**3.08**	0.90	0.39	**1.68**	**1.64**
KOR	2200	103.4	2.48	98.0	86.3	107.8	3.99	1.61	**1.36**	**1.47**	**1.30**	**1.16**	**1.71**
LTU	2265	93.3	2.07	95.6	90.8	101.5	2.29	1.11	0.65	0.35	**1.11**	**1.52**	0.02
LUX	2407	101.2	1.64	101.0	95.7	106.1	2.05	1.25	0.33	0.28	0.67	**1.33**	0.78
LVA	1751	84.1	2.08	83.0	73.3	88.5	3.33	1.60	0.85	**1.10**	0.23	**2.51**	0.87
MLT	1113	112.8	3.17	104.2	95.3	114.7	4.27	1.35	**2.16**	**1.35**	**2.96**	0.23	0.45
MNE	3066	89.2	1.57	84.3	78.2	92.4	2.84	1.81	0.97	0.35	**1.03**	**1.23**	**1.61**
MYS	2797	88.2	1.90	88.5	80.0	96.9	3.72	1.95	**1.44**	**1.42**	**1.04**	0.05	**2.19**
NLD	1787	90.0	2.54	90.2	78.7	101.5	5.55	2.19	**3.75**	0.31	0.22	**1.80**	**2.96**
NOR	2679	95.2	1.78	91.7	86.2	96.5	2.08	1.17	0.71	**1.10**	0.33	0.99	0.59
NZL	2821	97.9	1.64	99.4	95.9	103.4	1.79	1.09	0.36	0.05	0.37	**1.33**	0.43
POL	2577	94.2	2.12	95.4	89.7	99.3	1.94	0.92	**1.18**	0.87	0.14	0.70	0.75
PRT	2730	97.6	2.17	103.5	94.9	113.1	4.13	1.90	**3.23**	0.48	**1.21**	**1.90**	0.26
RUS	2510	84.6	2.16	85.7	81.0	93.0	2.59	1.20	**2.01**	**1.01**	0.24	0.38	0.30
SGP	2201	101.5	1.90	102.2	89.6	111.6	4.73	2.49	0.23	**1.78**	0.81	**1.09**	**3.92**
SVK	1904	97.8	2.26	99.2	92.0	109.8	3.06	1.35	0.71	**1.28**	0.73	**1.41**	0.96
SVN	2863	91.1	1.97	92.9	89.0	96.6	1.79	0.91	0.91	0.63	0.22	0.06	0.72
SWE	2539	95.1	1.89	97.0	89.3	103.3	3.23	1.71	**2.25**	**1.23**	0.84	0.88	0.20
TUR	3172	94.2	2.37	96.9	89.1	107.6	3.44	1.45	0.87	**2.14**	**1.27**	**1.21**	0.45
USA	2218	97.1	2.34	98.9	93.1	106.2	2.60	1.11	0.94	0.76	0.91	**1.39**	0.38

*Note*. cnt = country label (see Appendix A); N = sample size; M = composite estimator for multi-model inference (see (8)); ME = model error (see (9)); ER = error ratio defined as ME/SE (see (10)); Items = item choice (see Section 2.2.4); Model = specified IRT model (see Section 2.2.1); Pos = choice for handling position effects (see Section 2.2.5); RMSD = used cutoff value for RMSD item fit statistic for handling DIF (see Section 2.2.2); Score0 = scoring of missing item responses (see Section 2.2.3); Square roots of variance components larger than 1.00 are printed in bold.

## Data Availability

The PISA 2018 dataset is available from https://www.oecd.org/pisa/data/2018database/ (accessed on 16 May 2022).

## Abbreviations

The following abbreviations are used in this manuscript:

1PL	one-parameter logistic model
2PL	two-parameter logistic model
3PL	three-parameter logistic model
3PLRH	three-parameter logistic model with residual heterogeneity
ANOVA	analysis of variance
DIF	differential item functioning
ER	error ratio
IRF	item response function
IRT	item response theory
LSA	large-scale assessment
ME	model error
MML	marginal maximum likelihood
PIAAC	programme for the international assessment of adult competencies
PISA	programme for international student assessment
SE	standard error
SRVC	square root of variance component
TIMSS	trends in international mathematics and science study

## Appendix A. Country Labels for PISA 2018 Mathematics Study

The country labels used in the tables of the Results Section 3 are as follows:

ALB = Albania; AUS = Australia; AUT = Austria; BEL = Belgium; BIH = Bosnia and Herzegovina; BLR = Belarus; BRN = Brunei Darussalam; CAN = Canada; CHE = Switzerland; CZE = Czech Republic; DEU = Germany; DNK = Denmark; ESP = Spain; EST = Estonia; FIN = Finland; FRA = France; GBR = United Kingdom; GRC = Greece; HKG = Hong Kong; HRV = Croatia; HUN = Hungary; IRL = Ireland; ISL = Iceland; ISR = Israel; ITA = Italy; JPN = Japan; KOR = Korea; LTU = Lithuania; LUX = Luxembourg; LVA = Latvia; MLT = Malta; MNE = Montenegro; MYS = Malaysia; NLD = Netherlands; NOR = Norway; NZL = New Zealand; POL = Poland; PRT = Portugal; RUS = Russian Federation; SGP = Singapore; SVK = Slovak Republic; SVN = Slovenia; SWE = Sweden; TUR = Turkey; USA = United States.

## Appendix B. International Item Parameters for PISA 2018 Mathematics Study

Table A1 presents estimated item parameters for the international calibration sample 551 including 70 items if all missing item responses were treated as incorrect.

**Table A1:** Estimated item parameters for the 1PL, 2PL, and the 3PLRH model.

	1PL		2PL		3PLRH		
**Item**	a	$b_{i}$	$a_{i}$	$b_{i}$	$a_{i}$	$b_{i}$	$\delta_{i}$
CM033Q01S	1.273	−1.818	0.903	−1.615	0.656	−1.149	0.026
CM474Q01S	1.273	−0.951	0.924	−0.834	0.690	−0.668	0.763
DM155Q02C	1.273	−0.133	1.594	−0.113	1.045	−0.271	1.454
CM155Q01S	1.273	−0.999	1.482	−1.040	1.059	−0.864	1.142
DM155Q03C	1.273	2.259	1.357	2.318	0.962	1.643	−0.283
CM155Q04S	1.273	−0.176	0.995	−0.138	0.652	−0.220	1.091
CM411Q01S	1.273	0.072	1.683	0.119	1.090	−0.122	1.524
CM411Q02S	1.273	0.394	0.912	0.375	0.639	0.349	−0.832
CM803Q01S	1.273	1.482	1.918	1.824	1.282	1.205	0.626
CM442Q02S	1.273	1.223	1.940	1.528	1.296	0.971	0.769
DM462Q01C	1.273	3.612	1.413	3.726	1.010	2.623	−0.189
CM034Q01S	1.273	0.706	1.331	0.744	0.845	0.396	0.907
CM305Q01S	1.273	0.505	0.314	0.414	0.226	0.300	−0.155
CM496Q01S	1.273	0.240	1.500	0.287	1.025	0.125	0.506
CM496Q02S	1.273	−0.782	1.240	−0.771	0.881	−0.651	0.884
CM423Q01S	1.273	−1.489	0.833	−1.324	0.633	−0.974	0.393
CM192Q01S	1.273	0.541	1.428	0.601	0.991	0.459	−0.303
DM406Q01C	1.273	1.653	1.810	1.997	1.267	1.457	−0.824
DM406Q02C	1.273	2.575	2.595	3.802	1.865	2.743	−0.088
CM603Q01S	1.273	0.799	0.916	0.746	0.658	0.569	−0.416
CM571Q01S	1.273	0.374	1.376	0.416	0.955	0.342	−0.395
CM564Q01S	1.273	0.194	0.737	0.184	0.489	0.219	−0.988
CM564Q02S	1.273	0.275	0.718	0.253	0.455	0.295	−1.489
CM447Q01S	1.273	−0.638	1.440	−0.653	0.979	−0.392	−0.554
CM273Q01S	1.273	0.379	0.997	0.364	0.700	0.259	−0.036
CM408Q01S	1.273	0.885	1.290	0.921	0.850	0.557	0.680
CM420Q01S	1.273	0.118	1.041	0.125	0.715	0.023	0.481
CM446Q01S	1.273	−0.779	1.775	−0.886	1.264	−0.728	0.678
DM446Q02C	1.273	3.121	2.280	4.190	1.595	3.060	0.544
CM559Q01S	1.273	−0.458	0.876	−0.401	0.591	−0.241	−0.371
DM828Q02C	1.273	−0.498	1.082	−0.459	0.755	−0.446	1.053
CM828Q03S	1.273	1.154	1.271	1.185	0.768	0.699	1.038
CM464Q01S	1.273	1.545	2.006	2.001	1.389	1.379	0.280
CM800Q01S	1.273	−2.329	0.639	−1.988	0.711	−1.450	1.417
CM982Q01S	1.273	−2.075	0.922	−1.889	0.829	−1.407	1.387
CM982Q02S	1.273	0.995	0.977	0.912	0.603	0.552	0.725
CM982Q03S	1.273	−0.718	1.082	−0.673	0.772	−0.514	0.272
CM982Q04S	1.273	0.188	1.463	0.219	1.007	0.206	−0.426
CM992Q01S	1.273	−1.188	1.207	−1.164	0.792	−0.759	−0.530
CM992Q02S	1.273	2.333	1.846	2.779	1.291	1.961	−0.064
DM992Q03C	1.273	3.310	2.817	5.055	2.141	3.942	0.802
CM915Q01S	1.273	0.548	0.938	0.499	0.654	0.426	−0.718
CM915Q02S	1.273	−0.976	1.215	−0.956	0.889	−0.819	1.427
CM906Q01S	1.273	−0.485	1.233	−0.470	0.830	−0.283	−0.391
DM906Q02C	1.273	0.888	1.824	1.086	1.201	0.598	1.370
DM00KQ02C	1.273	2.551	1.166	2.464	0.883	1.763	−0.426
CM909Q01S	1.273	−2.383	1.710	−2.707	1.263	−1.941	0.322
CM909Q02S	1.273	−0.429	1.595	−0.455	1.110	−0.266	−0.520
CM909Q03S	1.273	1.024	2.379	1.445	1.677	0.927	0.760
CM949Q01S	1.273	−1.072	1.639	−1.183	1.177	−0.899	0.418
CM949Q02S	1.273	0.876	1.353	0.905	0.951	0.682	−0.447
DM949Q03C	1.273	1.093	1.456	1.160	1.000	0.785	0.177
CM00GQ01S	1.273	3.207	1.839	3.700	1.310	2.582	−0.430
DM955Q01C	1.273	−1.083	0.977	−0.978	0.735	−0.785	1.012
DM955Q02C	1.273	0.914	1.414	0.961	0.957	0.621	0.349
CM955Q03S	1.273	2.982	2.255	3.876	1.543	2.809	0.818
DM998Q02C	1.273	−0.854	1.185	−0.817	0.857	−0.655	0.614
CM998Q04S	1.273	0.690	0.236	0.529	0.264	0.414	−1.939
CM905Q01S	1.273	−1.436	1.020	−1.300	0.709	−0.908	−0.123
DM905Q02C	1.273	0.611	1.965	0.778	1.335	0.413	0.865
CM919Q01S	1.273	−1.781	1.672	−1.980	1.250	−1.490	1.185
CM919Q02S	1.273	0.391	1.106	0.384	0.654	0.110	1.327
CM954Q01S	1.273	−0.966	2.022	−1.177	1.456	−0.901	0.343
DM954Q02C	1.273	0.947	1.636	1.066	1.096	0.668	0.508
CM954Q04S	1.273	1.406	2.065	1.782	1.305	1.070	2.059
CM943Q01S	1.273	−0.053	0.855	−0.029	0.559	0.074	−0.930
CM943Q02S	1.273	3.979	2.474	5.277	1.723	3.909	0.478
DM953Q02C	1.273	0.690	1.435	0.735	0.982	0.469	0.273
CM953Q03S	1.273	0.052	2.007	0.098	1.394	−0.060	0.760
DM953Q04C	1.273	2.727	2.707	3.968	1.882	2.894	1.052

*Note*. 1PL = one-parameter logistic model; 2PL = two-parameter logistic model; 3PLRH = three-parameter logistic model with residual heterogeneity.
